# Resistin - the link between adipose tissue dysfunction and insulin resistance in patients with obstructive sleep apnea

**DOI:** 10.1186/2251-6581-12-5

**Published:** 2013-01-10

**Authors:** Radostina Vlaeva Cherneva, Ognian Borisov Georgiev, Daniela Stoichkova Petrova, Tsanko Lilianov Mondeshki, Sylvia Rumenova Ruseva, Adelina Dimitrova Cakova, Vanio Ivanov Mitev

**Affiliations:** 1 Department of Internal Medicine, Division of Pulmonary Medicine, Medical University, Sofia, Georgi Sofiiski 1str, Sofia 1431, Bulgaria; 2 Department of Medical Chemistry and Biochemistry, Laboratory of Synthesis and Analysis of Bioactive Substances, Medical University, Sofia, Zdrave 2str, Sofia 1431, Bulgaria

**Keywords:** Resistin, Insulin resistance, OSA, Diabetes, Normal glucose metabolism, Impaired glucose tolerance

## Abstract

**Background:**

Resistin is an adipocytokine, associated with obesity and inflammation. Its exact role in insulin resistance and diabetes in the general population is still controversial. The relation between resistin plasma levels, insulin resistance and risk of impaired glucose metabolism in OSA patients has not been investigated.

**Materials and methods:**

Plasma levels of resistin were measured in 67 patients with OSA and impaired glucose metabolism. 34,7% (23/67) had diabetes; 40% (27/67) patients had impаired glucose tolerance(IGT); 25,3%(17/67) had normal glucose metabolism (NGM). The association between resistin, BMI, obesity, markers of insulin resistance, oxidative stress and sleep study characteristics was analysed. The different groups of patients were compared in regards to glucometabolic parameters and biomarkers of oxidative stress – isoprostanes and insulin resistance – free fatty acids (FFA).

**Results:**

Plasma levels of resistin were higher in patients with diabetes (6,12 ±5,93ng/ml), compared to those with IGT (3,85±2,81ng/ml, p-0,021) and NGM (3,77±3,23, p-0,043). Resistin did not differ between patients with IGT and NGM (p-0,954). In OSA patients with BMI>40 resistin plasma levels correlated neither to the clinical parameters (BMI, IRI, HOMA-I, HbA1C, AHI, desaturation index), nor to the biomarkers of oxidative stress and insulin resistance. Free fatty acids (0,232>0,177mmol/l, p-0,037) were higher in diabetics in comparison to NGM.

**Conclusions:**

Plasma resistin levels in OSA patients with BMI>40 are independent of insulin resistance and are not associated with the parameters, characterising the oxidative stress or severity of OSA. Resistin could be used in a multiple panel of clinical and biomarkers to discern patients with diabetes from those with IGT; in OSA patients with BMI >40 resistin together with HbA1C could discern patients with diabetes from those with NGM. In OSA patients with BMI >40 FFA and HbA1C are useful clinical markers in assessing the risk of dysglycaemia among patients with normal and IGT.

## Introduction

Obstructive sleep apnea (OSA) is closely related to the metabolic syndrome. Systemic inflammation and oxidative stress have been thoroughly described as the main pathological triggers, provoking both metabolic and cardiovascular derangements in OSA patients. The inflammatory milieu is associated with the development of vascular injury in OSA [1]. Indeed, the degree of carotid atherosclerosis correlates positively with the respiratory disturbance index [2] and cytokine levels [3].

OSA is also closely related to insulin resistance which itself is a cardiovascular risk factor. Insulin resistance, oxidative stress and chronic inflammation coexist in OSA leaving the patients under increased risk for cardiovascular diseases.

In turn, the presence of OSA has emerged as an independent risk factor contributing to insulin resistance [4-6] and the metabolic syndrome [7,8]. The association between the metabolic consequences in OSA and inflammation have not been fully elucidated. Although a lot of basic research has been done, only a few can be implicated into clinical practice. Biomarkers that could be helpful for the early detection of insulin resistance and evaluating the level of oxidative stress could be helpful in the primary prevention of the cardiovascular consequences of OSA.

It is now clear that the routine biochemistry and the indexes, related to OSA severity are not the best indicators neither for the cardiovascular derangements, nor for the metabolic disruptions. This leads the door open for future research that is mostly clinically oriented providing a good study design as well as reasonable and pathophysiologically related biomarkers.

Resistin is an adipocyte-derived cytokine (adipokine) that may contribute to the development of obesity [9-12], insulin resistance [13,14] and the metabolic syndrome [15]. Recent studies have shown the causative association between resistin and systemic inflammation [16,17], especially in the vascular endothelium [18]. From the viewpoint of inflammation, it is notable that plasma resistin concentrations increase with increasing inflammatory mediator levels, predicting the severity of coronary atherosclerosis [19]. An investigation of resistin and its role for the generation of insulin resistance in OSA therefore provides a better understanding of complex links between metabolic disorders and cardiovascular involvements in this syndrome.A lot of controversial data has been generated, discussing its role in insulin resistance in the general population but not in OSA patients.

Free fatty acids have been used as predisposing factors for insulin resistance for a long time as well as a cardioavascular risk factor. In addition in OSA patients they are associated with sympathetic activity. Their role in insulin resistance in OSA patients is still elusive [20,21]. In contrast to these biomarkers isoprostanes are now generally assumed as reliable markers of oxidative stress, that could reflect the potential vascular damage a subject is exposed to [22,23].

The design of the study was conducted so that to find the role of resistin in the generation of insulin resistance, including subjects with newly found OSA. The primary aim was to investigate whether there is an incremental change of resistin from healthy to diabetic patients, analyzing its association with the clinical indicators – IRI, HOMA index; whether it refelects the oxidative stress - evaluated by the level of urinary isoprostanes, or is more closely related to insulin resistance and immunoreactive insulin. The secondary aim was to determine whether OSA severity, from normal to severe affects resistin levels.

We further evaluated the associations between resistin and lipid profiles and free fatty acids and tried to use them in a multipanel diagnostic model for the early detection of glucose impairment in OSA patients.

## Patients and methods

The protocol of the study was approved by the Ethics Committee of the University Hospital “Alexandrovska” and all the participants gave informed consent. We consecutively recruited 67 patients with newly defined OSA between June - December 2011. None of them had undergone any treatment for OSA. The definition of OSA was based on a combination of clinical symptoms (i.e. daytime excessive sleepiness) and a standard polysomnography.

After polysomnography, fasting blood samples were taken between 7 and 8 a.m. to examine plasma levels of resistin, and free fatty acids. A sample of overnight urine was taken to define the levels of urinary isoprostanes. Тhe biochemical tests were performed in the laboratory of synthesis and analysis of compounds, Department of Biochemistry, University Hospital “Alexandrovska”. Following admission, smoking habits (never, current or former smokers), medical history and regular medication use were recorded. Anthropometric data (height and weight) were recorded and BMI was then calculated as body weight (kg) divided by height squared (m^2^).

Daytime blood pressure was measured at least twice between 8 and 10 a.m. after 10 min of rest in the supine position. In all subjects, chest roentgenogram, electrocardiogram and pulmonary function test were performed. Routine blood examinations included: peripheral blood cell counts; hormones - TSH, FT3, FT4, morning and night cortisol; basic biochemistry - fasting plasma glucose, fasting serum insulin, creatinine, liver enzymes, fasting serum triglyceride, low density, very low density and high-density-lipoprotein cholesterol. Insulin resistance was calculated using the HOMA index: plasma glucose (mmol/l) x serum insulin( U/ml)/22,5 [24].

An oral glucose tolerant test was performed to define the patients with impairments in glucose metabolism. The test was performed as described by WHO [25] – subjects were fasting for at least 10 hours. After the sampling of fasting glucose they were loaded with 75g glucose. Blood glucose and IRI were measured within 2 hours. Impaired fasting glucose was defined as fasting glucose – 6,1-7mmol/l; impaired glucose tolerance – as blood glucose in the range 7,8-11,1mmol/l two hours after the glucose burden; diabetes – fasting blood glucose >7mmol/l at least twice or random blood glucose >11,1mmol/l. All laboratory tests were performed in the Central Clinical Laboratory, University Hospital “Alexandrovska”.

Exclusion criteria were as follows: 1) age > 80 years; 2) current prescription of anti-inflammatory drugs, statins, steroids; 3) the presence of any of the following medical conditions: chronic kidney diseases, chronic respiratory failure, primary heart diseases, endocrine disorders or neoplasm.

### Polysomnography

Polysomnographic parameters were continuously recorded via a computerized data acquisition system Compumedics, E-series. The following parameters were documented:sleep stage (electroencephalogram and electro-occulogram), electrocardiogram, nasal airflow, thoracic and abdominal movements and oxyhemoglobin saturation (SpO2). Apnea was defined as complete cessation of nasal airflow for at least 10s. Hypopnea was defined as a > 50% reduction in nasal airflow lasting at least 10s, with oxygen desaturation > 3%. The AHI was calculated as the average number of apnea plus hypopnea events per hour of sleep. The degree of nocturnal hypoxia was assessed as: 1) the percentage of the total sleep time during which SpO2 decreased below 90% (time SpO 2 < 90%).

### Laboratory assays

Blood samples were centrifuged immediately after collection and isolated plasma was stored in vials at −80°C until assayed. Resistin and free fatty acids were measured by commercial kits, following the procedure protocol.

Resistin was determined by an ELISA kit (RayBio_ Human Resistin ELISA Kit Protocol (Cat#:ELH-Resistin-001) The intra- and interassay coefficients of variation in this assay kit ranged from 10 to 12%. Plasma resistin levels were measured in ng/ml.

The concentration of free fatty acids was determined by an enzymatic colorimetric method assay for the quantification of non-estserified free fatty acids – (WAKO NEFA-HR2). Results are given in mmol/l.

### HRAM determination of 8-isoprostane in urine samples

The levels of 8-isoprostane in urine samples were determined by HRAM (high resolution accurate mass) mass spectrometry on LTQ Orbitrap® Discovery (ThetmoScientific Co, USA) mass spectrometer, equipped with Surveyor® Plus HPLC system and IonMax® electrospray ionization module. The analyses were carried out by stable isotope dilution method in negative ionization mode using HESI II (heated electrospray ionization) source type.

The concentration and purification of 8-isoprostane from urine samples was processed by affinity sorbent (Cayman Chemical, USA), following the producer’s protocol with some modification. Briefly, approximately 1 ml of urine sample was centrifuged for 10 min at 4°C and 13500 rmp. Then, to 300 μl of supernatant was added 700 μl of phosphate buffer (0.1M, pH=7.4), 10 μl internal standard (D4-8-isoprostane, 0.1 ng/ml in H_2_O) and 110 μl affinity sorbent. The sample was incubated for 45 min at room temperature. After that the sample was centrifuged for 15 min at 4°C, 13500 rmp and supernatant was carefully removed by decanting. The sample was washed once with 1 ml of phosphate buffer (0.1 M, pH=7.4), centrifuged for 15 min at 4°C, 13500 rmp and the supernatant was carefully removed. After that the same washing step was repeated with 1 ml of ultra pure water. The 8-isoprostane was eluted from the affinity sorbent by 2 x 0.5 ml of 95% ethanol. The samples were evaporated to dryness under vacuum and reconstructed in 150 μl mixture of water-acetonitrile (70:30, v/v).

Chromatographic separation was carried out on XTerra C18 column (I.D. 2.1 mm x 150 mm, 3,5 μm particle size, Waters, USA) using as eluents: A-0.1% Formic acid in water; B - 0.1% Formic acid in acetonitrile at flow rate of 250 μl/min. The followed binary gradient was used: 30–90% B for 10 min; 90–30% B for 1 min; and 30% B for 4 min.

The HRAM spectra in range of m/z 200–700 were acquired on Orbitrap® mass analyzer set to operates at 30 000 resolution. The all Orbitrap MS parameters were optimized for sensitivity to the target analyte using the instrument control software program. The below presented parameters were used: spray voltage 3.2 kV; Spray current 100 μA; Sheath gas flow rate 40; Auxiliary gas flow rate 10; Sweep gas flow rate 0; Capillary voltage −37 V, Capillary temperature 280°C; Vaporizer temperature 250°C and Tube lens −99.07 V. All data acquisition and processing were carried out with XCalibur® software package (ThetmoScientific Co, USA. The ions with m/z = 353.238 and m/z =357.263, were used to monitor 8-isoprostane and D4-8-isoprostane, respectively. The linear calibration curve was established using a hundred picograms of D4-8-isoprostane for internal standard and the external standard of 8-isoprostane in range of 100 pg/ml to 5000 pg/ml. Five levels of calibration standards were measured at the beginning and in the end of the unknown samples. Two levels of quality control samples were injected after every 20 samples to monitor the inter- and intraday accuracy and precision.

The urinary isoprostane levels were standardized to the levels of urinary creatinine. It was measured applying the enzyme method - Creatinine plus version 2 Cobas Integra (Roche). Results are given in pg/mkmol/creatinine.

### Statistical analysis

Statistical analysis was performed using SPSS (version 14.0; SPSS) A p<0,05 was considered of statistical significance. Continuous variables are expressed as means ±SD. Kolmogorov-Smirnov was used to find if normal distribution existed. Comparison between the three groups of OSA patients (diabetics, IGT, NGM) was made using one way analysis of variance (ANOVA) if normal distribution or Kruskall-Wallis if non-parametric data was found. Mann-Whithney test was performed to compare two groups of patients according to different variables. Correlations between paired variables were performed using Pearson (normal data) or Spearman (nonparametric data) correlation analysis.

## Results

Patients were divided into three groups according to their glucose metabolism – patients with already known diabetes – 23; patients with impaired glucose tolerance – 27; patients with normal glucose metabolism – 17. Anthropometric, glucometabolic, sleep study and biological characteristics are given in Table 1.

**Table 1:** Patients’ characteristics in the study group

	**Diabetic patients (23)**	**Impaired glucose tolerance (27)**	**Normal glucose metabolism (17)**
Anthropometric characteristics			
Age, years	56.95±10.38	49.76±9.04	42.56±7.49
			**p-0.671**
M/F	17/6	23/4	17/0
BMI, kg/m^2^	42.86±7.29	40.36±9.37	43.11±8.6
			**p-0.785**
Waist circumference, cm	133.2±20.84	130.12±15.96	138.14±19.76
			**p-0.421**
**Sleep study characteristics**			
Mild to moderate OSA	2/23(9%)	11/27(40%)	2/17 (12%)
Severe OSA	21/23 (91%)	16/27 (60%)	15/17 (88%)
AHI, events/h	58.79±34.25	53.00±35.33	62.33±30.58
			**p-0.671**
Sleep duration, min	194.03±56.81	192.87±55.04	236.41±59.11
			**p-0.09**
Time with SpO2 <90%,	70.85±25.99	56.03±33.44	49.75±32.4
			**p-0.114**
**Lipid profiles**			
High density Chol, mmol/l	1.27±0.31	1.27±0.35	1.29±0.26
			**p**-**0.982**
Low density Chol, mmol/l	2.86±0.07	3.03±0.093	3.24±1.03
			**p-0.448**
Very low density Chol, mmol/l	0.83±0.09	0.85±0.07	0.83±0.32
			**p-0.980**
Tot Chol, mmol/l	4.97±1.02	5.19±1.17	5.25±1.04
			**p-0.623**
Trigl, mmol/l	1.71±0.82	2.03±0.84	2.07±0.82
			**p-0.385**
**Insulin resistance**			
Fasting glucose, mmol/l	6.99 ±1.33	5.96±1.71	4.89 ±0.71
Immunoreactive insulin, mU/l	18.92±16.57	31.06±28.05	22.65±12.45
	p*-0.006	p**-0.229	p***-0.151
HOMA index	4.86±4.46	7.48±7.66	5.24±3.33
			p***-0.177
HbA1C	6.69±1.11	6.41±1.06	5.63±0.44
	p*-0.253	p**-0.003	p***-0.000
**Biomarkers**			
Resistin, ng/ml	6.12±5.93	3.85±2.81	3.77±3.23
	p*-0.021	p**-0.954	p***-0.043
Free fatty acids, mmol/l	0.267±0.146	0.232±0.088	0.180±0.071
	p*-0.78	p**-0.121	p***-0.037
Isoprostanes	0.113±0.09	0.07±0.06	0.07±0.05
			**p-0.134**

**p**- Kruskall –Wallis comparison between three groups; p*- Mann -Whithney comparison diabetics vs IGT; p**- Mann -Whithney comparison IGT vs NGM; p***- Mann -Whithney comparison diabetics vs NGM.

### Anthropometric, clinical and biological characteristics in the three groups, regarding the impairments in their glucose metabolism

Regarding the anthropometric and sleep study characteristics no large discrepancies existed between groups. They were similar according to the age, BMI, smoking status, gender distribution. No difference was established, when analyzing their sleep study parameters. The lipid profiles did not differ in regards to any of the fractions investigated.

The analysis of the indicators related to the glucose metabolism showed a statistically significant difference between the plasma levels of the immunoreactive insulin in patients with IGT in comparison to those with diabetes – 31,06±28,05 vs.18,92±16,57 (p −0.006). The HOMA – index did not differ much between the groups. The glycated haemoglobin however was statistically higher in patients with IGT - 6,41±1,06 vs. those with normal glucose metabolism 5,63±0,44 p-0.003. The same trend could be found when the diabetics and the patients with normal glucose metabolism were compared - 6,69±1,11 vs 5,63±0,44 (p-0,000). Resistin plasma levels were significantly higher in patients with diabetes - 6.12±5.93 in comparison to those with normal glucose metabolism - 3.77±3.23, p-0.043. Similar are the results when diabetics and patients with impaired glucose metabolism are compared – p −0.021. The significant difference remains even after adjustment for age, BMI and HOMA-I.

### Clinical and biological characteristics in the groups, regarding the severity of OSA

Based on the AHI, OSA patients were subdivided into mild-to-moderate (AHI = 5–30 events/h) OSA group – 19; and severe (AHI > 30 events/h) OSA group - 48. Patients’ characteristics are given in Table 2. The severity of OSA influences neither the lipid, nor the glucometabolic profiles. No difference occurred between the two groups regarding their biological parameters –Table 2.

**Table 2:** Clinical and biological markers in the groups, regarding the severity of OSA

***Biomarkers***	**Mild-to-moderate OSA (19)**	**Severe OSA (48)**
Resistin, ng/ml	3.96±2.97	4.92±4.8
		**p-0.362**
Free fatty acids mmol/l	0.213±0.08	0.241±0.12
		**p-0.375**
Isoprostanes	0.089±0.07	0.088±0.08
		**p-0.985**
***Lipid profiles***		
High density Chol, mmol/l	1.37±0.21	1.23±0.32
		**p-0.821**
Low density Chol, mmol/l	3.05±0.81	3.01±0.95
		**p-0.872**
Very low density Chol, mmol/l	0.84±0.36	0.83±0.43
		**p-0.952**
Tot Chol, mmol/l	5.31±1.09	5.06±1.08
		**p-0.405**
Trigl, mmol/l	1.92±0.82	1.91±0.99
		**p-0.786**
***Insulin resistance***		
Immunoreactive insulin, mU/L	20.47±10.06	26.31±24.5
		**p-0.470**
HOMA index	5.04±3.69	6.35±6,45
		**p-0.586**
HbA1C	6.32±0.9	6.31±1.09
		**p-0.681**

### Association between resistin plasma levels anthropometric, clinical and biological markers in OSA patients

The analysis of the relationship between resistin plasma resistin levels and anthropometric, clinical, biological and sleep apnea parameters, cigarette consumption is shown in Table 3. Resistin plasma levels did not correlate to any of them.

**Table 3:** Correlation between resistin plasma levels, anthropometric, clinical and biological markers in OSA patients

	**Correlation level**
**Anthropometric characteristics**	
Age, years	**p-0.629**
M/F	**p-0,439**
BMI, kg/m^2^	**p-0.902**
Waist circumference, cm	**p-0.376**
Smokers (current/nonsmoker)	**p-0.622**
**Sleep study characteristics**	
AHI, events/h	**p-0.619**
Sleep duration, min	**p-0.988**
Time with SpO2 <90%,	**p-0.284**
**Lipid profiles**	
High density Chol, mmol/l	**p**-**0.931**
Low density Chol, mmol/l	**p-0.473**
Very low density Chol, mmol/l	**p-0.760**
Tot Chol, mmol/l	**p-0.631**
Trigl, mmol/l	**p-0.376**
**Insulin resistance**	
Fasting glucose, mmol/l	**p-0.376**
Immunoreactive insulin, mU/l	**p-0.583**
HOMA index	**p-0.694**
HbA1C	**p-0.849**
**Biomarkers**	
Free fatty acids, mmol/l	**p-0.870**
Isoprostanes, pg	**p-0.134**

#### Clinical implication of the investigated markers

**Figure 1:**
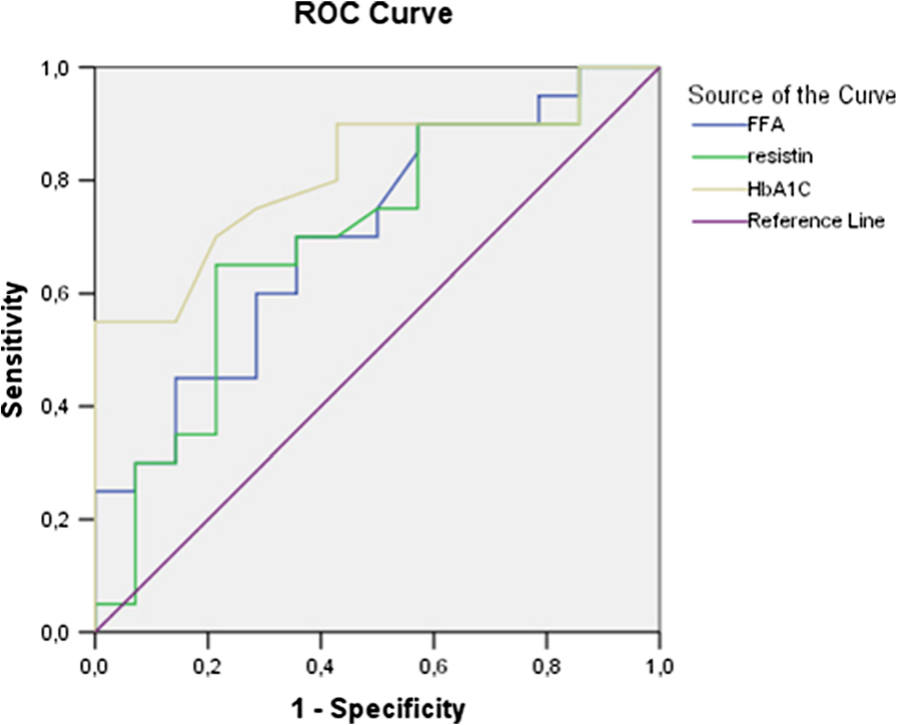
ROC analysis of FFA, resistin and HbA1c curves in patients with diabetes and normal glucose tolerance.

ROC analysis was performed to analyse the clinical utility of the biomarkers that differed significantly between groups. The ROC analysis of FFA, resistin and HbA1C among diabetic and healthy patients is presented in Table 4, (Figure 1).

**Table 4:** ROC analysis in diabetic/normal glucose metabolism patients

	**Area under the curve**	**P - value**	**95% CI – lower bound**	**95% CI - upper bound**
**FFA,mmol/l**	0.692	0.042	0.527	0.857
**Resistin, ng/ml**	0.689	0.044	0.518	0.859
**HbA1c**	0.838	0.064	0.713	0.963

**Figure 2:**
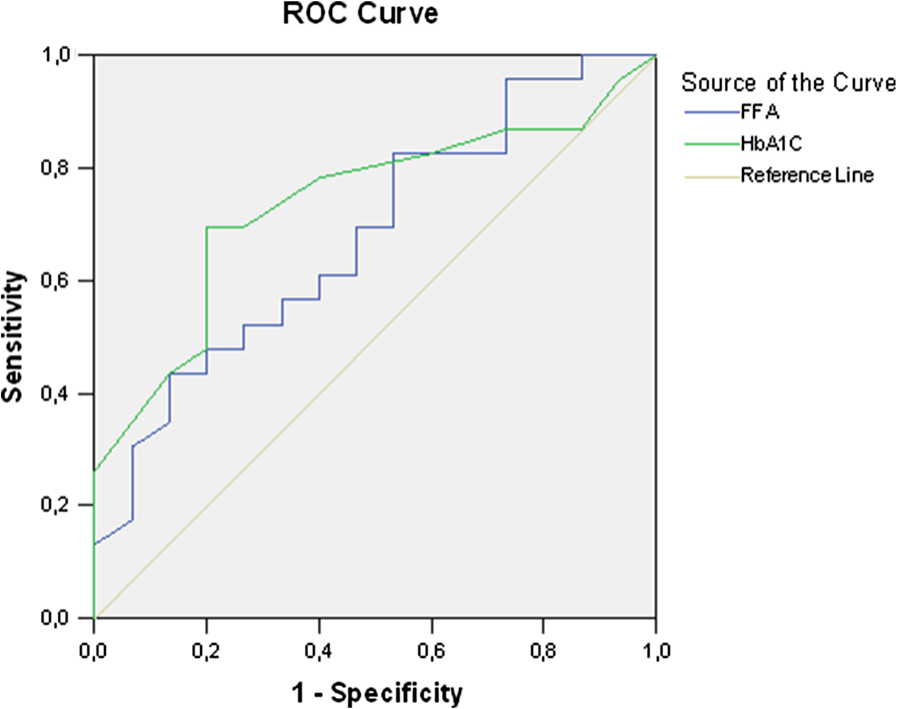
ROC analysis of FFA and HbA1c curves in patients with impaired glucose tolerance and normal glucose metabolism.

The ROC analysis shows that resistin plasma levels of 3,45ng/ml had sensitivity - 68% and specificity – 69% in distinguishing diabetic from healthy patients; FFA at a cut-off of 0,211 mmol/l has sensitivity 61% and specificity – 67%; HbA1c – 5,7% seems to be with the best sensitivity (78%) and specificity (75%) as a diagnostic marker. Similar are the results when comparing patients with impaired glucose tolerance and healthy ones. ROC analysis could be performed in regards to FFA and HbA1c. Data is presented in Table 5. (Figure 2). The specificity (63%) and sensitivity (80%) of the well validated diagnostic marker - HbA1c – 5,7% is better than that of FFA - specificity – 64% / sensitivity 70% at a cut-off – 0,170mmol/l.

**Table 5:** ROC analysis in impaired glucose tolerance/normal glucose metabolism patients

	**Area under the curve**	**P - value**	**95% CI – lower bound**	**95% CI - upper boundary**
**FFA,mmol/l**	0.678	0.065	0.506	0.850
**HbA1c**	0.769	0.073	0.625	0.913

ROC analysis shows that in OSA diabetic patients with high grade obesity the validated by the American Diabetes Association diagnostic value of HbA1c – 6,4% is less specific and sensitive, compared to 5,7%. Similar are the results regarding OSA patients with impaired glucose tolerance and high grade obesity - HbA1c – 5,7% is better than the range 5,7-6,4%.

**Figure 3:**
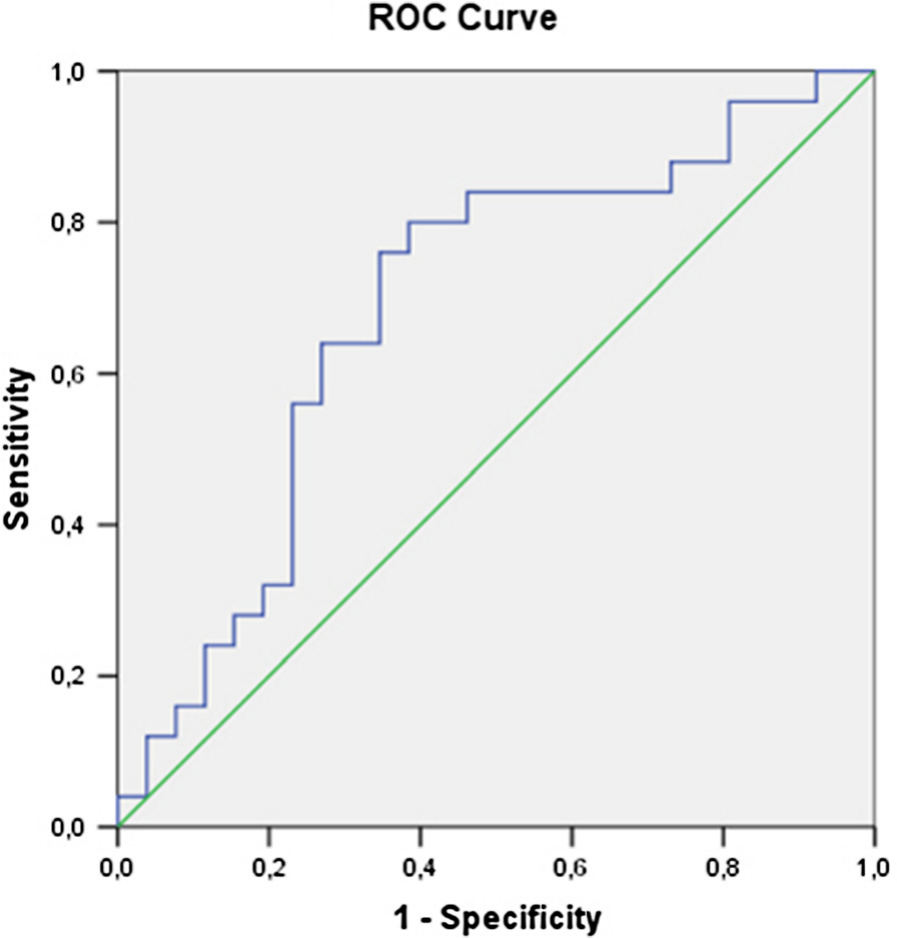
ROC analysis of resistin in patients with diabetes and inpaired glucoe tolerance.

The only biomarker that differed between diabetic and impaired glucose tolerance patients was resistin At plasma levels of 4,03ng/ml it shows sensitivity - 64% and specificity – 67% in distinguishing diabetic from impaired glucose tolerance patients Table 6 (Figure 3).

**Table 6:** ROC analysis in impaired glucose tolerance/normal glucose metabolism patients

	**Area under the curve**	**P - value**	**95% CI – lower bound**	**95% CI - upper boundary**
**Resistin, ng/ml**	0.688	0.022	0.538	0.838

## Discussion

Patients with sleep apnea commonly develop the metabolic syndrome, the hallmark of which is insulin resistance. It appears to be one of the major risk factors for atherosclerotic and cardiovascular diseases [26]. The mechanisms by which obesity and intermittent hypoxia predispose to insulin resistance in that patient group are of interest.

Adipose tissue dysfunction that is characterized by the imbalanced secretion of different adipokines has been largely disputed in clinical studies. Adiponectin and leptin are adipose-specific hormones that improve glucose tolerance. Both have been thoroughly investigated in different studies. Several other adipose-derived hormones oppose insulin action, and they circulate at increased levels in obesity, thus also possibly contributing to insulin resistance. Prominent among them are tumor necrosis factor-α and interleukin-6, two cytokines that are also produced at high levels in macrophages and were first identified as inflammatory mediators [27,28]. Results are rather controversial to shed light and promote the implementation of a certain biomarker neither for screening of impaired glucose tolerance in OSA, nor as a factor for stratification of patients at high risk of developing metabolic syndrome.

Resistin is a 12-kDa polypeptide that was initially linked to insulin resistance in animal models [29]. Early reports suggested that resistin is associated with obesity and insulin resistance in rodents. Deletion of the resistin gene reduces the impact of obesity on glucose homeostasis [30]. Conversely, acute administration of resistin impairs glucose tolerance and insulin action [31]. Mice with chronic hyperresistinemia exhibit modest fasting hyperglycemia and glucose intolerance, associated with increased hepatic glucose production in the setting of hyperinsulinemia. These results indicate that chronic hyperresistinemia leads to impairment of glucose homeostasis [32].

A number of studies have examined plasma resistin levels or adipose resistin expression, and have found variable associations with insulin resistance [11,33-35]. A recent large study involving the Framingham offspring cohort found a significant relationship between insulin resistance and resistin. This relationship however was considerably weaker than the relationship with adiponectin, and was lost after adjustment for BMI [36]. Resistin decreases after TZD treatment of humans, although resistin was also decreased by metformin treatment [19]. Therefore, resistin is clearly an important adipokine that likely plays a role in the development of insulin resistance.

An investigation of resistin in OSA will therefore provide a better understanding of the complex interrelation between metabolic disorders, inflammation and cardiovascular involvement.

According to the results of our study there is not a significant difference between the plasma levels of resistin in patients with impaired and normal glucose tolerance. The two groups were comparable regarding the age, BMI, waist circumference and the severity of OSA. The duration of sleep, the average desaturation index and the time spent under SpO2 <90% also did not differ much between the groups.

The analysis of resistin plasma levels on the other hand showed correlation neither to the anthropometric (age, BMI, waist circumference, smoking status), nor to the sleep study or glucometabolic characteristics. The other biomarkers, indicative of insulin resistance and oxidative stress also turned to play no role on resistin plasma levels. Assuming this data it seems that resistin alone is not a trigger of insulin resistance in obese OSA patients. It more likely plays a secondary role in the complex adipokine signaling, accompanying adipose tissue dysfunction.

Our results support those of Yamamoto et al. [37], who find no association between resistin plasma levels and insulin resistance, presented by the should be HOMA-I in OSA patients. Their study however should be consciously reconsidered as it is performed in Asian population, with significantly lower BMI (BMI-28). Wysozka et al., [38] investigated different adipokines in Caucasian obese (BMI 30–39,9) and overweight (BMI – 25–29,9) patients with and without OSA. They conclude that in both groups – obese and overweight - OSA per se causes a decrease in resistin plasma levels.

Having this in mind and considering that each of the three groups we studied consisted of extremely obese patients with an average BMI >40 (only 10% have BMI 30–35) we can speculate that the secretion of resistin could be blunted. If true this makes it rather difficult to detect the slight difference in resistin plasma levels between OSA patients with IGT and NGM if any exists.

Wysoczka et al. [38], describe that in both obese groups – with and without OSA resistin correlated to an increased fasting glucose. Similar is the data, presented by Rangwala et al. [32]. They detect that under chronic hyperresistinaemia normal weight and on normal diet mice have high fasting glucose. This they prove is due to increased hepatic gluconeogenesis. Moreover impaired fasting glucose persists in the model despite the adaptive hormonal rearrangements that take place. In our study however we can not find such a relation in none of the studied groups.

We found no association between high levels of insulin and HOMA-I and resistin neither in patients with normal, nor in those with IGT. This is in controversy to what is reported in obese patients (BMI-33) without diabetes [39]. Again it is very probable to assume that extreme OSA per se blunts resistin secretion.

In human adipose tissue, resistin seems to be produced mainly by infiltrating macrophages [17].

Recent studies have shown the causative association between resistin and systemic inflammation [18], especially in the vascular endothelium [40].It is notable that plasma resistin concentrations increase with increasing inflammatory mediator levels, predicting the severity of atherosclerosis [41]. These observations detected in the general population are also confirmed in OSA patients by Yamamoto and Lee. The data presented by Yamamato et al., [37] shows that plasma resistin levels increase with the severity of OSA. This correlates best with AHI and is associated to increased inflammation, reflected by the higher concentrations of IL-6. Similar are the findings of Lee et al. [34]. They also demonstrate that plasma resistin levels increase with the severity of OSA and that this trend correlates best to AHI. We should take in mind that both studies are performed in Asian population which deters the application of their findings in Caucasians. In pediatric OSA however where much of the confounding factors are abolished the increase in plasma resistin levels with the severity of AHI is very persuasive [42].

In our study we find a tendency for an increase of resistin with the severity of OSA that is not statistically significant. The plasma levels of the marker in the group with moderate apnea, compared to those with severe one are respectively – 3,92vs4,96ng/ml, not reaching statistical significance. A reason for this can be that our patients were extremely obese – the average BMI >40. In the study of Yamamoto et al. [37], and Lee et al. [34], the average BMI is 28. Even though the distinction in the levels of resistin are best remarked when comparison is made between patients with severe OSA and the control group (p<0,01). Plasma resistin levels between moderate and severe OSA even in slight degrees of obesity – (BMI – 28) do not reach statistically important difference. The influence of the intermittent hypoxia, accompanying OSA, can therefore be obscured in extremely obese patients.

### Limitations

The limitations of our study are that: First patients were of similar age and BMI, but extremely obese, which complicates the applicability of data to the general OSA population; Second patients were predominantly men as a gender dimorphism is present in plasma resistin [9,43] the results can not be translated to women with OSA.

Last but not least we performed a cross-sectional study, encompassing a relatively small number of subjects with various impairments of glucose metabolism, which does not allow the establishment of any causality, but only the propositions of certain hypothesis about the role of resistin in the metabolic derangements in OSA.

## Conclusions

In conclusion according to the results of our study resistin plasma levels in OSA patients with BMI>40 do not correlate to any of the clinical parameters - anthropometric, glucometabolic, lipid or sleep study characteristics of the patients. Resistin plasma levels were higher in patients with diabetes, compared to those with IGT and NGM. In OSA patients with BMI>40 resistin did not differ between patients with IGT and NGM and did not change significantly with the severity of OSA. This shows that resistin alone could not be an indicator of insulin resistance in OSA patients, at least not in extremely obese one. It could however be a part of a multiple diagnostic panel. In OSA diabetic patients with high grade obesity HbA1c – 5,7% is of better diagnostic value than the validated – 6,4%.

## Abbreviations

OSA: Obstructive Sleep Apnea; AHI: Apnea+Hypopnea Index; BiPAP: Bilevel Positive Airway Pressure; BMI: Body Mass Index; HOMA-I: Homeostasis Model Assessment Index; IRI: Immuno Reactive Insulin; OGTT: Oral Glucose Tolerance Test; RR: Riva Rocchi; FFA: Free Fatty Acids; IGT: Impaired Glucose Tolerance; NGM: Normal Glucose Metabolism.

## Competing interests

The authors declare that they have no competing interests.

## Authors’ contributions

RCh, OG, VM and DP participated in the design of the study and in writing the manuscript. RCh recruited the patients and collected the data. TM,OG performed the PSG studies. SSR and VL performed the HRAM analysis and free fatty acids measurements. AC performed the ELISA for resistin and the enzymatic determination of urinary creatinine levels. All reviewed and approved the final version of the manuscript.
